# A Multilingual, Culturally Competent Mobile Health Intervention to Improve Treatment Adherence Among Women Living With HIV: Protocol for a Randomized Controlled Trial

**DOI:** 10.2196/17656

**Published:** 2020-06-19

**Authors:** Lunthita M Duthely, Alex P Sanchez-Covarrubias, Adhar B Mohamed, JoNell E Potter

**Affiliations:** 1 Obstetrics, Gynecology and Reproductive Services Miller School of Medicine University of Miami Miami, FL United States; 2 Miami Clinical and Translational Sciences Institute Miller School of Medicine University of Miami Miami, FL United States

**Keywords:** telemedicine, HIV, acquired immunodeficiency syndrome, women, adherence, clinical trial protocol, barriers, facilitators, text messaging, mHealth, SMS/texting

## Abstract

**Background:**

Adherence to HIV care is complex, as barriers to care are multidimensional, particularly for ethnic minority women. Mobile health (mHealth) solutions are supportive in improving HIV health care outcomes. In the United States, however, mHealth interventions are not widely implemented in public HIV clinics and have not been customized for women. There is an unmet need for culturally and linguistically appropriate mHealth interventions that address the health care needs of minority women living with HIV.

**Objective:**

This study aims to describe a protocol investigating the feasibility of an mHealth intervention for treatment adherence among women living with HIV. This is a two-phase, mixed methods, pilot randomized controlled trial that begins with qualitative patient interviews to inform the system design. Participants will be block randomized by language (English, Spanish, and Haitian Creole) to 1 of 2 study arms.

**Methods:**

Women (age ≥18 years) who were followed up at the women’s HIV clinic of an academic medical center, with a recent history of nonadherence to HIV care (missed appointments, unsuppressed viral load, or not taking medications as prescribed), will be enrolled. The experimental arm will receive the intervention, which includes health reminders and psychoeducational messaging, plus clinical standard of care reminders. The psychoeducational messaging will target patient-level barriers of HIV stigma and medical mistrust and resilience as a patient-level strength. The control arm will receive standard of care reminders only (ie, mailed appointments and automated telephone calls). All aspects of the study and intervention will be offered in the participants’ preferred language. The primary outcome is the feasibility and acceptability of the study. The secondary outcomes are changes in self-reported medication adherence, depression symptoms, HIV stigma, medical mistrust, resilience, and clinic attendance and viral suppression extracted from the participants’ medical records. Data will be assessed at baseline (T0) and 2 subsequent clinic visits—approximately 3 to 4 months from the baseline (time 1; T1) and 6 to 9 months from the baseline (time 2; T2). Qualitative data will be transcribed and analyzed iteratively. Bivariate analyses will compare data by the study group (chi-square, odds ratios, and t tests). Exploratory analyses will be conducted for each outcome variable—T1 and T2 values will be compared with values at T0 by the study group.

**Results:**

As of March 2020, baseline quantitative data were collected on 54 participants (28 English speakers, 14 Spanish speakers, and 12 Haitian Creole speakers). The first 3 focus groups (1 in each of the 3 languages) were completed, with a total of 20 participants. The findings are currently being integrated into the beta version of the mHealth texting system.

**Conclusions:**

The findings of this novel HIV adherence intervention may shed light on the barriers and facilitators of HIV health care and the mechanisms of an mHealth intervention that is customized for ethnic minority women living with HIV.

**Trial Registration:**

ClinicalTrials.gov NCT03738410; https://clinicaltrials.gov/ct2/show/NCT03738410

**International Registered Report Identifier (IRRID):**

DERR1-10.2196/17656

## Introduction

### Background

Adherence to care (eg, visit adherence) is the single most important factor projected to improve health outcomes for persons living with HIV and reducing communal HIV viral load, relieving both the individual and public health burden of HIV [1]. Adherence to care is complex, as barriers are multidimensional. Barriers are experienced at the individual or patient level (ie, mental health and psychosocial) or can occur as a result of structural issues (ie, insurance coverage) [2]. In the United States, barriers to regular HIV care disproportionally affect women and ethnic minorities [3,4]. Metropolitan Miami has the highest HIV transmission rate in the United States; however, only 61% of women living with HIV are engaged in HIV care [5,6]. In Miami, a multicultural and multilingual region, two-thirds of the residents are foreign born and speak a language other than English. Specifically, 60% of the residents speak Spanish or Haitian Creole at home [7]. Mobile health (mHealth) technology, such as texting, has shown promise in improving HIV care adherence, both globally and domestically, in a variety of settings [8].

### Rationale

mHealth solutions, which range from texting to full-blown apps, are supportive in improving HIV health outcomes [8]; however, in the United States, where 95% of the residents own a mobile phone, mHealth is not widely implemented in public HIV clinics. Furthermore, most mHealth interventions in the United States for HIV care adherence are designed for specific populations such as sexual minority men or adolescents. Interventions tested among ethnic or linguistic minorities have focused on a single ethnic group and were monolingual. Furthermore, only a few have been designed for women [8]. Women access health care at specific life stages—pregnancy, primary, and gynecological care; these stages are opportunities to engage women in HIV care and address barriers to regular care. Among women, the frequency of engaging in care depends on their life stage. Postnatal women, who may be adequately engaged in HIV care, for example, fall out of care after the birth of their child [9]. Effective solutions need to reach all sectors of the population. Taken together, there is an unmet need for culturally and linguistically appropriate mHealth interventions that address the health care needs of women living with HIV.

Individual barriers to HIV care adherence are often interrelated and affect individuals differently. In the literature, HIV stigma (eg, discrimination experienced because of an HIV diagnosis) and medical mistrust (ie, distrust of providers or a medical system) often co-occur—a combination that directly affects HIV health outcomes among minorities [2]. In a recent study, medication mistrust was found to be directly related to HIV care adherence among African Americans [10]. HIV stigma is also known to be related to depression [2]. In examining strengths or coping mechanisms, resilience, which is defined as adaptation in the face of adversity, mitigates the negative barriers to positive health outcomes for minority women living with HIV [11].

In a public clinic in Miami, serving a predominantly minority population, a significant correlation was demonstrated between HIV care barriers and HIV care adherence. Depression and low patient-physician relationship ratings correlated significantly with low adherence. Moreover, patients who endured multiple barriers were more likely to have detectable viral loads [12]. HIV stigma, medical mistrust, and depression can be used to measure barriers to health care access. The relationship among these previously identified barriers, combined with a person’s resilience, may inform interventions to improve HIV care adherence.

### Objective

The overall goal of this innovative intervention is to develop a patient-centered mHealth system tailored to individual patients that addresses their unique HIV barriers. The system will deliver different text messages and will be designed for a cohort of multicultural and multilingual women living with HIV, who were followed up at a large, public academic medical center in Metropolitan Miami.

### Study Comparators

The control arm for this study are enrolled participants who will receive the hospital’s standard of care, as it relates to the reminders for clinical appointments. The hospital system mails reminder letters 7 to 10 days before the appointment. Patients also receive automated reminder telephone calls, approximately 2 working days before the appointment.

### Theoretical Foundation

**Figure 1:**
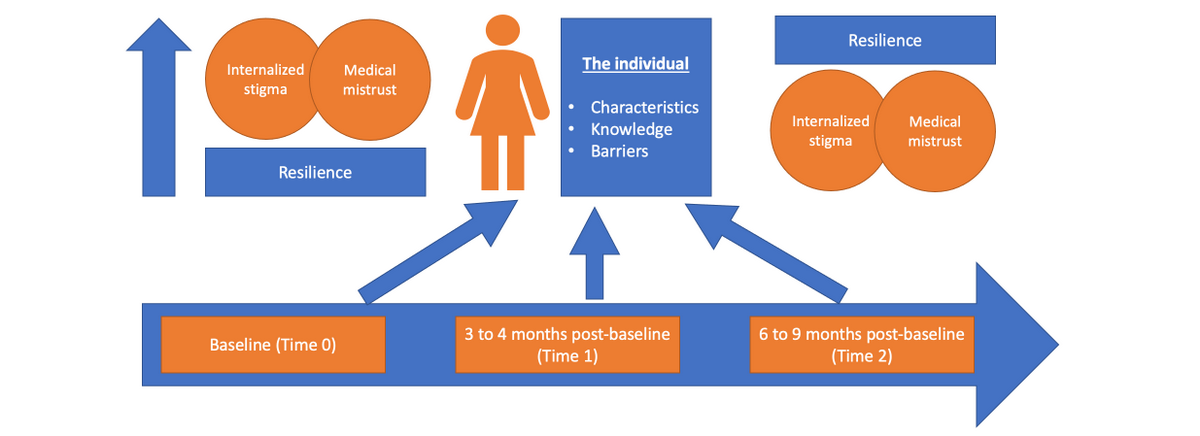
Protocol conceptual framework based on the Health Belief Model.

Conceptually, the design of the intervention is consistent with the Health Belief Model (HBM) [13,14]. The HBM considers individual-level characteristics, strengths, and barriers to influence health behavior change. The mHealth system includes health reminders and psychoeducational (behavioral) messaging to reduce nonadherence, internalized stigma (one’s own negative perceptions about HIV), and medical mistrust while strengthening resilience. Behavioral messaging is adapted from the Dale and Safren’s Striving Towards Empowerment and Medication Adherence (STEP-AD) intervention, which aims to reduce nonadherence and the experiences of stigma and to improve resilience for women living with HIV [15]. The conceptual framework of the protocol is depicted in Figure 1.

### Trial Design

This is a concurrent, two-phase, mixed method, pilot randomized controlled trial. Both the qualitative and quantitative components collect data from the target population women living with HIV. The trial begins with the qualitative component.

#### Phase 1

The qualitative component comprises interviews in the form of participant focus groups and participant key informant sessions.

#### Phase 2

The quantitative component is a short-term longitudinal intervention, where participants will be randomized to 1 of 2 study arms (parallel group). The allocation ratio is set at 1:1. The control arm will receive the standard of care for reminders, and the experimental arm will receive the intervention, in addition to the standard of care for reminders (see Study Comparators section). Data will be collected at baseline (T0), 3 to 4 months postbaseline (time 1; T1), and 6 to 9 months postbaseline (time 2; T2) after the launch of the intervention (exploratory trial).

## Methods

### Study Setting and Population

The study site is a women’s HIV clinic, located within an academic medical center in Metropolitan Miami, United States. Participants will be enrolled from various services of the clinic, including prenatal care, primary care, and gynecological specialty care.

### Eligibility Criteria

The following are the eligibility criteria for the study: (1) women aged ≥18 years; (2) confirmed HIV diagnosis (per the clinic’s standard); (3) active patient (ie, not withdrawn, transferred, or dismissed); (4) 2 or more previously scheduled visits 12 months before the study enrollment; and (5) nonadherence, based on 1 or more of the following criteria: missed 1 or more visits, having a viral load >20 copies/mL, or not taking HIV antiretroviral medications as prescribed.

Participants will have to confirm at the time of enrollment that they possess a working mobile phone number (to receive text messages). We will exclude women with serious psychiatric diagnoses, who are cognitively impaired or are not able to consent for themselves.

### Study Consent Process

Participants will give consent in person with the institutional review board (IRB)–approved paper copies of forms and an ink signature. First, clinic providers and staff will inform the patients of the study, including the purpose of the study and participation criteria. Second, patients who express interest in participating will be referred to a research team member who will be on site to provide further information. Potential candidates for the study will be read the informed consent form and the Health Insurance Portability and Accountability Act (HIPAA) form in their preferred language (English, Spanish, or Haitian Creole). A trained and IRB-approved study team member will explain the different aspects of the study in simple words and answer the study candidates’ questions. Interested candidates will then be asked 3 to 4 questions to assess the understanding of the project, purpose, procedures involved, and the voluntary nature of participation. Those who cannot successfully answer the questions will have the study re-explained by the research staff, with an opportunity for clarification on the areas that they did not understand. Only participants demonstrating an understanding of the study and voluntary agreement to participate will be invited to sign the forms. To accommodate low written literacy in the participants’ respective languages (Spanish or Haitian Creole), bilingual study team members will be on hand to ensure that the participants understand the questionnaires.

Before the beginning of each focus group, participants will be rebriefed on the purpose of the study and informed that the focus group will be recorded. Moreover, the participants will be informed of their right to remove themselves from the study or withdraw their responses at any time, without penalty. The respondents will also have the opportunity to ask questions before the session begins.

### Intervention Components

#### Qualitative Component

The qualitative component comprises 6 focus groups of 5 to 8 participants in each of the 3 different languages (English, Spanish, and Haitian Creole). Overall, 3 focus groups (focus groups before baseline; FG1), 1 per language, will occur before T0—the launch of the intervention. The second set of focus groups (focus groups at the end of the intervention; FG2) will occur at the end of the intervention (T2). The FG1s, conducted before T0, will inform the customization of the mHealth system; the FG2s, conducted after T2, will elicit the participants’ experiences with the intervention. The focus group participants will also complete the quantitative questions described in Table 1.

**Table 1:** Summary of assessments and data collected for primary and secondary study outcomes.

Study outcomes			Study measures: description		Variable type and values		Data source (time point)	
**Primary outcomes**								
	**Rates of enrollment**							
				The proportion of participants enrolled out of the total number of participants approached. The proportion of participants who declined enrollment or failed the screening out of the total number of participants approached		Continuous; range 0-1		Study logs (T0^a^)
	**Acceptability of the mobile health app**							
				The proportion of messages opened out of the number of messages received		Continuous; range 0-1		Data usage logs (T0, T1^b^, and T2^c^)
**Secondary outcomes**								
	**Clinical outcomes**							
		Adherence to medication		Wilson 3-item self-report measure for medication adherence (self-administered) [16]		Continuous (score); range 1-100		Participant (T0, T1, and T2)
		Clinic attendance (change)		Number of missed visits 1 year before assessment (T0), between T0 and T1, and between T1 and T2		Continuous (number)		Electronic medical record
		Viral suppression		Viral load		Categorical; ≥20 copies/mL or <20 copies/mL		Electronic medical record or measured through a blood test (T0, T1, and T2)
	**Behavioral outcomes**							
		Depressive symptoms		Patient health questionnaire-9 (self-administered) [17-19]		Continuous (score); range 0-27		Participant (T0, T1, and T2)
		Internalized stigma		HIV stigma scale (self-administered) [20,21]		Continuous (score); range 40-160		Participant (T0, T1, and T2)
		Medical mistrust		12-Item group-based medical mistrust scale (self-administered) [22,23]		Continuous (score); range 12-60		Participant (T0, T1, and T2)
		Resilience		25-item Connor-Davidson resilience scale (self-administered) [24-26]		Continuous (score); range 0-100		Participant (T0, T1, and T2)

^a^T0: baseline.

^b^T1: time 1 (3 to 4 months postbaseline).

^c^T2: time 2 (6 to 9 months postbaseline).

The qualitative component will also comprise 6 sets of key informants, where 2 to 3 participants will represent each language. The first group of key informants (KI1) will be drawn from the FG1, approximately 1 month after FG1 for each language. KI1s will be invited to be interviewed based on their interaction with the mHealth system. The second group of key informants (KI2), who will be sampled from the intervention group, will be interviewed after T1. KI1 and KI2 will provide interim feedback to fine-tune the design of the mHealth system.

#### Quantitative Component

##### Mobile Health Messaging System Prototype: Delivery of Text Messages

An English-version prototype of the mHealth messaging system using commercially available software for mobile texting has been designed. A basic user front end populates the system with data from an Excel (Microsoft Corp) spreadsheet, including mobile phone numbers, message types (medication reminder or behavioral messages), message frequency (weekly or daily), start/stop data, and message content. The communication is bidirectional, in that participants can respond with preselected codes (eg, *1* and *2*), responding to prompts such as “Press ‘1’ if you received this message.”

##### Mobile Health Messaging System Prototype: Privacy and Security

The system will not transmit personal health information (PHI). Exact names of clinics or terms such as “HIV” or “AIDS” will not be transmitted. The patients’ phone numbers are the only identifiers that will be stored in the system. Twilio is HIPAA, International Organization for Standardization 27001, and General Data Protection Regulation Privacy Shield compliant. Twilio is password-protected, and programmers use a two-factor authentication process to access the programming environment.

#### Connection Between the Qualitative and Quantitative Components

**Figure 2:**
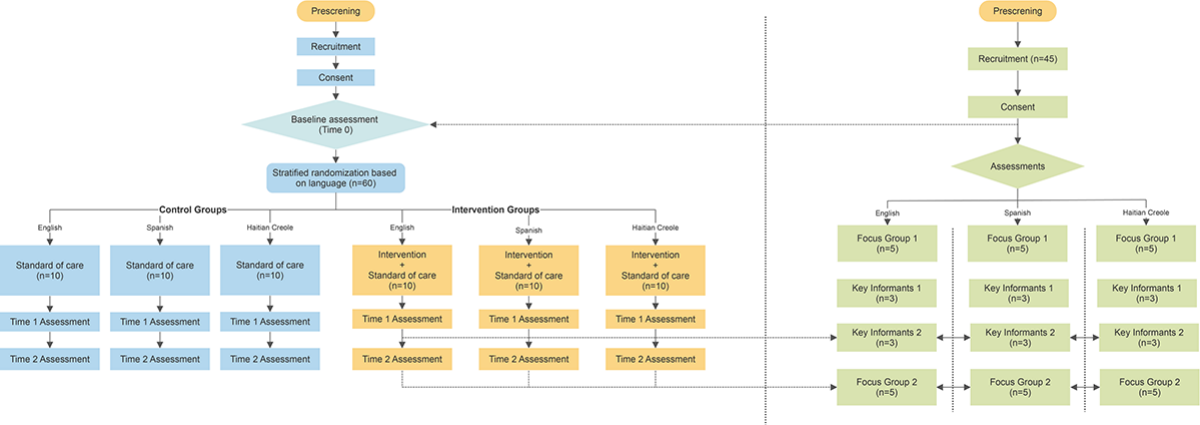
Data flow diagram for the study quantitative components (left) and the qualitative components (right).

This is a parallel, mixed methods study, where the results from the qualitative component will inform the design of the intervention (quantitative component). As depicted in Figure 2, the qualitative and quantitative components will connect at 3 time points: before T0, at T1, and T2 (study end). FG1, KI1, and KI2 will provide insights into the messaging preference (reminder and behavioral) for the customization and refinement of the mHealth prototype and also pilot the mHealth prototype. All messaging will be piloted with focus groups by language.

### Intervention: Personalization of Reminders and Behavioral Messages

Text messages set for medication reminders will be customized based on timing, frequency, and exact content per participant preference. Behavioral messages address the barriers of internalized stigma, medical mistrust, and resilience at the individual/personal level. Participants will be blinded to their survey results. The baseline scores of participants (high internalized stigma, high medical mistrust, or low resilience) will determine the behavioral message content. At T0, participants will provide their customizations for the system. At T1 or T2, and any other time the participants choose, updates will be entered into the system. Behavioral messages have been adapted from the STEP-AD intervention aimed at decreasing internalized stigma and medical mistrust while improving adherence and resilience for women living with HIV. Suggested text messages will be presented to participants at FG1, conducted in the 3 different languages, to test for acceptability in the population of interest.

### Intervention Fidelity

#### Laboratory Data

If participants miss their scheduled clinical appointment, they will be asked to come to the clinic’s research wing, where a blood sample will be drawn by a clinical research staff member to obtain the viral load. Quantitative data will be collected as well. This procedure will ensure adherence to study data points T1 and T2.

#### Texting System

For the mHealth system, participants will be prompted to respond to predefined responses such as “Press ‘1’ if you received this message” and “Press ‘2’ if you would like us to call you.” Downloadable data usage logs will track messages that are opened and the participants’ responses. Periodically, participants will receive text surveys regarding the usefulness of a message immediately upon opening the message. These data will further guide messaging development and gauge intervention adherence.

### Intervention: Concomitant Care

At the discretion of the principal investigator (PI), participants who are concurrently enrolled in another intervention with overlapping objectives may be excluded from the data analysis. The proposed intervention will supplement the standard of care provided by the hospital, as it relates to appointment reminders.

### Study Outcomes

The primary and secondary outcomes are summarized in Table 1. Data will be collected either from the electronic medical record (EMR), the HIV database, or directly from the participants’ responses to questionnaires. For analysis, all outcomes (Table 1) will be reported as a change from baseline. As this is a pilot study, our intention is to determine the most sensitive measures. We will explore the data to determine the most suitable categorizations for a future study; therefore, the total scores will be reduced to categories once all data have been compiled. To report on the participant characteristics, sociodemographic data, including age, race/ethnicity, HIV or AIDS status, socioeconomic status, pregnancy status, substance use, and mental health history (past year), will be collected at T0.

Clinical outcomes (at T0, T1, and T2) that will be examined include a comparative history of self-reported medication adherence, visit attendance, and viral suppression reported as the biological marker of HIV viral load (copies/mL). Psychological and behavioral data include depressive symptoms, internalized stigma, resilience, and medical mistrust (Table 1).

### Power Analysis

This study aims to develop and measure the feasibility of a pilot system. Therefore, a power analysis is not appropriate for this study. The plan is to estimate the expected effect sizes and determine the sensitivity of the measures at the conclusion of the study.

### Target Sample Size

For the qualitative component, approximately 15 participants per language will be enrolled to ensure that 5 to 7 participants (per language) will convene for the FG1; therefore, a total of 45 participants will be enrolled to prepare for FG1. As a feasibility trial, this study is descriptive in nature, with the ability to test the study hypotheses. For the quantitative component, approximately 20 participants will be recruited per language, of which half will be randomized to the control group.

### Recruitment Strategy

Participants will be enrolled from a women’s HIV service in an academic medical center where approximately 900 individual women are seen annually. An estimated 50 patients are seen weekly. The women’s HIV service is made up of a variety of clinics, including prenatal, primary care, and gynecological specialty care. The women’s HIV service collects sociodemographic information, appointments, and laboratory/pathology results in its database (Ryan White Care Ware system) to comply with the reporting and quality requirements of its various funding sources. Information such as missed appointments, adherence to medication, viral load, primary language spoken, and ethnic/racial background will be extracted from the Ryan White Care Ware system for all potential study participants with a confirmed appointment.

### Assignment to Study Arms

Participants will be randomized 1:1 to 1 of the 2 treatment arms. Randomization will be stratified by language, where approximately 10 participants per language group will be enrolled in each study arm. This ensures that approximately equal numbers of participants by language groups are represented in each study arm. A randomization schedule will be created by the study statistician based on the Excel function of random number generation. The randomization schedule will be of a randomized block nature to ensure relative equality of assignment across conditions and to prevent the potential of study staff guessing the next assignment. Randomization will take place upon the completion of baseline assessments. The study group assignment will be communicated back to the study team member who consented the patient within 24 hours of enrollment.

### Data Collection, Management, and Analysis

#### Data Collection Plan

##### Qualitative Component

Audio recordings gathered from all qualitative data (FG1, FG2, KI1, and KI2) will be transcribed using a commercially available transcription software available in English and Spanish. In addition, 2 study team members will review the entire transcription and make changes manually through the software. The Haitian Creole audio will be transcribed manually. The transcripts, along with other information (see Instrumentation), will be entered into a software package designed for mixed methods analyses [27].

##### Quantitative Component

Unless stated otherwise, all data will be collected at T0, T1, and T2. Participants will complete their paper forms. All questionnaires are meant to be self-administered, but a research team member will be present at all times to answer any questions that the participant may have.

##### Quantitative Data Sources

Data will be collected from the clinic’s EMR, the women’s HIV service database, and directly from the participants.

#### Instrumentation

##### Sociodemographic, Clinical, and Behavioral Data

Demographic, behavioral and mental health history, and clinical data outcomes, including viral suppression and visit history, will be extracted from the EMRs for each participant. Demographic data will be collected at the time of consent for both the qualitative and quantitative studies.

##### Background, Financial Information, and Viral Suppression (Self-Reported)

Self-reported sociodemographic information, including age, race/ethnicity, place of birth, marital status, relationship status, highest level of education completed, employment status, annual income, adherence to medication, viral load, and CD4 count, will be collected.

##### Three-Item Self-Report Measure for Medication Adherence

Medication adherence will be measured using the 3-item self-report measure for medication adherence, which examines *days taken*, *frequency*, and *rating of medications* over the past 30 days. This questionnaire has been validated using an electronic drug monitoring device (Cronbach α=.84 for HIV antiretroviral medications) [16]. The questionnaire was translated into Spanish and Haitian Creole and will be piloted with FG1 participants.

##### HIV Stigma Scale

Internalized stigma will be measured using the HIV-related stigma scale, a 40-item measure validated in both English (Cronbach α=.96) [20] and Spanish (Cronbach α=.91) [21]. Individuals rate statements such as “Some people act as if it’s my fault I have HIV” on a 4-point Likert-type scale (*1=strongly disagree* to *4=strongly agree*). The scale will be translated into Haitian Creole and then piloted with the FG1.

##### Group-Based Medical Mistrust Scale

Medical mistrust will be measured by the group-based medical mistrust scale, which comprises 12 items (Cronbach α=.64-.70), with statements such as “I have personally been treated poorly or unfairly by doctors or health care workers because of my ethnicity” rated by patients on a 5-point Likert-type scale (*1=do not agree at all* to *5=completely agree*) [22]. It has been translated and validated into Spanish (Cronbach α=.80) [23]. A Haitian Creole version has been created and will be piloted with FG1 participants.

##### Resilience

The Connor-Davidson resilience scale (CDRISC-25) is a validated 25-item questionnaire (Cronbach α=.89), including statements such as “When things look hopeless, I don’t give up,” and responded to on a 5-point Likert-type scale (*0=not true at all* to *4=true nearly all of the time*) [24]. The CDRISC-25 has been translated and validated in Spanish (Cronbach α=.89) [25] and in Haitian Creole (Cronbach α=.80) [26].

##### Patient Health Questionnaire-9

Depressive symptoms will be measured using the Patient Health Questionnaire-9. Participants will rate the questionnaire on a 3-point Likert-type scale based on how often they have been bothered by any of the symptoms stated (*0=not at all* to *3=nearly every day*). It has been validated in English (Cronbach α=.89) [17], Spanish (Cronbach α>.89) [28], and Haitian Creole (Cronbach α=.78) [19].

#### Data Management

The completed questionnaires will be stored in a secured cabinet in a locked room inside the PI’s office. Only the PI and study team members will have access to the data forms and electronic versions of the collected data. Data will be extracted from the paper surveys and entered into an HIPAA-compliant database. Deidentified data will be exported into file formats appropriate for analysis. Authorized team members will use their unique password-protected accounts to access the database. A master spreadsheet will be available to all study team members, uploaded into a *cloud* document manager, and the participant’s study ID number will be recorded. This spreadsheet will be encrypted and will be the only database that contains identifying information. All data collection forms, consent forms, and the study log will be stored for 3 years after the closure of the study. The audio and transcription files will also be stored in the *cloud* document manager.

### Study Outcomes

#### Qualitative Data Analyses

Recordings from FG1, FG2, KI1, and KI2, for both the English and Spanish languages, will be processed using a commercially available transcription software and reviewed by a study team member fluent in that language. Haitian Creole will be transcribed manually. All transcriptions will be reviewed a second time by a different study team member fluent in the language. Spanish and Haitian Creole language texts will be first translated into English. The English texts will be analyzed iteratively for thematic content (major themes). Themes pertaining directly to the research questions will inform the mHealth system customization. Dedoose [27] mixed methods analysis software will facilitate data analysis. Overall, 2 study team members will review, enhance, and categorize the software-generated codes. A codebook will document each iteration of the data analysis.

#### Quantitative Data Analysis

##### Data Preparation

###### Sociodemographic and Clinical Data

Participants’ mobile numbers will be matched to the mHealth texting system logs; participants’ medical record number (MRN) will be matched to the Ryan White Care Ware database and the hospital’s EMR. Inconsistent data and outliers will be reviewed. Missing data and unresolved data discrepancies will be considered and recorded as *missing data* and excluded from the analyses.

###### Study Measures

Questionnaires will be evaluated and compared with the expected response ranges. Participant-level records with incomplete responses will be considered missing for all instruments.

##### Descriptive Statistics

Groups (intervention vs control) will be compared by baseline characteristics to detect significant group differences, (eg, chi-square values and odds ratios for frequency tables and *t* tests for continuous variables). Cutoffs at *P*<.05 will determine 2-tailed significance. If needed, an alternate (Fisher exact test) or a nonparametric analysis will be conducted.

##### Exploratory Analyses

We will conduct exploratory analyses, where analysis of variance (ANOVA) models will be constructed for each outcome variable. Statistically significant baseline characteristics, mHealth usage, clinically relevant characteristics, and total instrument score will be entered as covariates. The ANOVAs will compare T1 and T2 outcomes to values at T0 by the study group. At T1 and T2, clinical and behavioral outcomes will be compared with values at T0 for both study arms.

##### Statistical Analysis: Missing Data

For individual statistical tests, only records with complete data will be included in the analyses.

#### Data, Safety, and Confidentiality

##### Data and Safety Monitoring Plan

A data and safety monitoring plan was developed and registered with the funding agency. We anticipate that the largest risk in this study is a breach of confidentiality. Breaches of confidentiality will be reported to the IRB, where action at the institutional level will take place. In addition, the study procedures will be stopped, and we will re-evaluate our procedures.

##### Potential Harms

Potential unexpected events include a breach in confidentiality, as some participants would not have disclosed their HIV status to their intimate partners, family members, or friends. Some participants may be sharing their mobile devices, and so a potential risk exists. Adverse events and serious adverse events may be reported spontaneously by the participant. If a potential adverse event is identified, the PI will report the adverse event directly to the IRB. Events that are psychosocial in nature and that meet the criteria for reporting to the IRB (unexpected, related, and serious) will be reported in the 10-day timeframe established. Other events will be reported annually.

##### Confidentiality

A breach of confidentiality is still a possible risk, but measures will be taken to minimize this risk. The study team will have to collect names and contact information to schedule a participant for a focus group or contact as a key informant and conduct the chart review; therefore, complete anonymity is not possible.

Participants will be assigned an ID number, and only the ID number will identify the data collected on all questionnaires. The questionnaires will not be linked to the consent forms and contact information forms and will not record any identifying information such as name, date of birth, or contact information. The only protected health information that will be stored in the mHealth system is the participants’ cell phone numbers. No PHI will be transmitted to the participants. Only the study team members will have access to the data. A study log, which includes MRN, study identification number, and date consented, will be kept in an electronic file, secured with an encrypted password. Only the study team members will know the passwords. The contact information forms and recordings will be destroyed upon the completion of the study. The consent forms and logs will be destroyed after 3 years. The participants’ names or personal identifiers will never be transmitted or be part of the study publication of its findings.

The participants’ audio recordings, study records, and survey results will be kept private and will not be identified in any publication. Data will be presented in aggregate only. The completed questionnaires, contact information forms, consent forms, and recording devices will be kept in a locked cabinet in the office of the PI. The data collection form, along with the electronic files of the focus group and key informant recordings and their transcriptions will be stored in a university-approved HIPAA-compliant database and in cloud-based storage. Only the study team members will have access to the data. Data collection forms and transcriptions will be kept for analysis. The completed questionnaires will be destroyed after 3 years.

#### Research Ethics Approval

The PI obtained IRB approval to conduct the study. The IRB approved the study personnel, consent forms, recruitment materials, instruments, and all other materials given to the participants. Changes to the study protocol will be submitted in writing to the IRB, and only IRB-approved modifications will be implemented.

## Results

The study was funded in December 2018. IRB approval for the English language version of the protocol was attained in January 2019. As of March 2020, baseline quantitative data have been collected on 54 study participants (28 English speakers, 14 Spanish speakers, and 12 Haitian Creole speakers). The first 3 focus groups (1 in each of the 3 languages) have taken place, with a total of 20 participants. The findings from these data are currently being integrated into the design of beta version of the mHealth texting system.

## Discussion

### Overview

As of April 2020, 54 participants have been enrolled. Based on the number of subjects enrolled, we anticipate continued participation and enrollment, where we will further test the system and launch the intervention.

### Strengths and Limitations

The most significant strength of this study is that, to our knowledge, it is the first HIV adherence intervention in the United States that is designed for minority women across multiple cultural and linguistic contexts. We recognize that the most significant limitation of the study is that there may not be sufficient power to test complex interaction models for the quantitative arm of the analyses. Furthermore, because the intervention is being tested among a cohort of women with a history of nonadherence, study retention may be negatively impacted.

### Conclusions

This study aims to determine the feasibility of a mobile intervention on adherence to HIV health care among ethnic minority women living with HIV across multiple linguistic and cultural contexts. The beta version of the mHealth texting system is currently being designed. One limitation is that the study may be underpowered for higher order statistical analyses of the quantitative findings. Nevertheless, the proposed intervention is unique, and the findings of the study may shed light on the barriers, facilitators, and mechanisms of a novel intervention customized for the needs of ethnic minority women living with HIV. Furthermore, the findings of this study were carried out successfully and will serve as the basis for a clinical trial with a larger sample size to fully test the intervention’s effectiveness.

## Appendix

Multimedia Appendix 1 Peer review reports from Miami CTSI KL2 Awards FY19.

## Abbreviations

ANOVA: analysis of variance
CDRISC-25: Connor-Davidson Resilience scale
EMR: electronic medical record
HBM: Health Belief Model
HIPAA: Health Insurance Portability and Accountability Act
IRB: institutional review board
mHealth: mobile health
MRN: medical record number
PHI: personal health information
PI: principal investigator
STEP-AD: Striving Towards Empowerment and Medication Adherence

## References

[1] Shah M, Perry A, Risher K, Kapoor S, Grey J, Sharma A, Rosenberg ES, del Rio C, Sullivan P, Dowdy DW (2016). Effect of the US national HIV/AIDS strategy targets for improved HIV care engagement: a modelling study. Lancet HIV.

[2] Earnshaw VA, Bogart LM, Dovidio JF, Williams DR (2013). Stigma and racial/ethnic HIV disparities: moving toward resilience. Am Psychol.

[3] (2017). Centers for Disease Control and Prevention.

[4] (2016). Centers for Disease Control and Prevention.

[5] (2017). Centers for Disease Control and Prevention.

[6] (2018). Centers for Disease Control and Prevention.

[7] (2018). Miami-Dade Beacon Council.

[8] Henny KD, Wilkes AL, McDonald CM, Denson DJ, Neumann MS (2018). A rapid review of ehealth interventions addressing the continuum of HIV care (2007-2017). AIDS Behav.

[9] Momplaisir FM, Storm D, Nkwihoreze H, Jayeola O, Jemmott JB (2018). Improving postpartum retention in care for women living with HIV in the United States. AIDS.

[10] Kalichman SC, Eaton L, Kalichman MO, Cherry C (2017). Medication beliefs mediate the association between medical mistrust and antiretroviral adherence among African Americans living with HIV/AIDS. J Health Psychol.

[11] Dale S, Cohen M, Weber K, Cruise R, Kelso G, Brody L (2014). Abuse and resilience in relation to HAART medication adherence and HIV viral load among women with HIV in the United States. AIDS Patient Care STDS.

[12] Wawrzyniak AJ, Rodríguez AE, Falcon AE, Chakrabarti A, Parra A, Park J, Mercogliano K, Villamizar K, Kolber MA, Feaster DJ, Metsch LR (2015). Association of individual and systemic barriers to optimal medical care in people living with HIV/AIDS in Miami-Dade County. J Acquir Immune Defic Syndr.

[13] Glanz K, Rimer B, Viswanath K (2008). Health Behavior and Health Education: Theory, Research, and Practice.

[14] Rosenstock IM (1974). Historical origins of the health belief model. Health Educ Monogr.

[15] Dale SK, Safren SA (2018). Striving towards empowerment and medication adherence (STEP-AD): a tailored cognitive behavioral treatment approach for black women living with HIV. Cogn Behav Pract.

[16] Wilson IB, Lee Y, Michaud J, Fowler FJ, Rogers WH (2016). Validation of a new three-item self-report measure for medication adherence. AIDS Behav.

[17] Kroenke K, Spitzer RL, Williams JB (2001). The PHQ-9: validity of a brief depression severity measure. J Gen Intern Med.

[18] Wulsin L, Somoza E, Heck J (2002). The feasibility of using the Spanish PHQ-9 to screen for depression in primary care in Honduras. Prim Care Companion J Clin Psychiatry.

[19] Marc LG, Henderson WR, Desrosiers A, Testa MA, Jean SE, Akom EE (2014). Reliability and validity of the Haitian Creole PHQ-9. J Gen Intern Med.

[20] Berger BE, Ferrans CE, Lashley FR (2001). Measuring stigma in people with HIV: psychometric assessment of the HIV stigma scale. Res Nurs Health.

[21] Jimenez JC, Puig M, Ramos JC, Morales M, Asencio G, Sala AC, Castro E, Santori CV, Santiago L, Zorrilla C (2010). Measuring HIV felt stigma: a culturally adapted scale targeting PLWHA in Puerto Rico. AIDS Care.

[22] Thompson HS, Valdimarsdottir HB, Winkel G, Jandorf L, Redd W (2004). The group-based medical mistrust scale: psychometric properties and association with breast cancer screening. Prev Med.

[23] López-Cevallos DF, Harvey SM, Warren JT (2014). Medical mistrust, perceived discrimination, and satisfaction with health care among young-adult rural latinos. J Rural Health.

[24] Connor KM, Davidson JR (2003). Development of a new resilience scale: the Connor-Davidson resilience scale (CD-RISC). Depress Anxiety.

[25] Manzano-García G, Calvo JC (2013). Psychometric properties of Connor-Davidson resilience scale in a Spanish sample of entrepreneurs. Psicothema.

[26] Blanc J, Rahill GJ, Laconi S, Mouchenik Y (2016). Religious beliefs, PTSD, depression and resilience in survivors of the 2010 Haiti earthquake. J Affect Disord.

[27] (2019). Dedoose.

[28] Familiar I, Ortiz-Panozo E, Hall B, Vieitez I, Romieu I, Lopez-Ridaura R, Lajous M (2015). Factor structure of the Spanish version of the patient health questionnaire-9 in Mexican women. Int J Methods Psychiatr Res.

