# Supplementary material for: A Multilingual, Culturally Competent Mobile Health Intervention to Improve Treatment Adherence Among Women Living With HIV: Protocol for a Randomized Controlled Trial
Source: JMIR Res Protoc. 2020 Jun 19;9(6):e17656. doi: 10.2196/17656 (PMC7334759; doi:10.2196/17656)
Supplement: Multimedia Appendix 1 [file resprot_v9i6e17656_app1.pdf]

CTSI KL2 Awards FY19 - Reviewer Evaluation Form

Dear Miami CTSI KL2 Reviewer,

Thank you for accepting to review this proposal.

Please submit your reviews by September 17, 2018.

If you have any question regarding this award, or the evaluation form, please contact ...

The RFA and Instructions are available on the CTSI website:
http://www.miamictsi.org/researchers/funding-opportunities/ctsi-k12-program-awards

Best regards,

The CTSI Team

Response was added on 09/10/2018 1:41pm.

CONFLICT OF INTEREST

The following are considered potential conflicts for reviewing grant proposals:
• You have a financial and/or professional relationship with the applicant
• The funding decision on any application would benefit or affect you directly
• You feel there may be a perception of conflict
• You are a current collaborator, or have collaborated/published with the applicant in the last two years.

Note: You may review applications within the same department as long as none of the above conditions apply.

By checking the box below, you confirm that you do not have any conflict of interest in reviewing this proposal

☒ I confirm

SECTION 1: REVIEWER'S INFORMATION

SECTION 2: Miami CTSI KL2 AWARD APPLICANT INFORMATION

Applicant's Name (Last,

First) ....

Department

...

...

Collaborator(s) on this project

P...

Proposal Title

Multi-Lingual, Culturally-Copetent mobile Health Intervention to Reduce Medical Mistrust, Stigma and Improve Treatment Adherence among Women Living with HIV/AIDS

SECTION 3: REVIEW

The focus of the KL2 award program of the Miami CTSI is to help outstanding early career investigators establish academic careers involving important translational, transformational research programs examining disease processes or outcomes related to health disparities.

Prior to proceed with this review, please read carefully the review criteria for the Miami CTSI KL2 Awards listed below:
• Compliance with all eligibility requirements (the administrative review is completed)
• Commitment and potential for a career in clinical and/or translational research.
• Commitment of the necessary resources and protected time from the primary department.
• Scholarship, as assessed from biosketch, letters of reference, and past academic performance.
• Evaluation of the proposed research project (scientific merit, potential clinical importance, scholar's ability to execute the research plan, available resources, likeliness of successful completion).
• Evaluation of the proposed mentored development plan, appropriateness and commitment of mentoring team and collaborators.
• Likelihood that the KL2 support will contribute to a successful K or R Award application (or equivalent).
• The impact of the research on improving the health of minority and underserved population.

The CTSI KL2 FY19 RFA and Instructions are attached here as one PDF.

[Attachment: "KL2 FY19 RFA & Instructions.pdf"]

A. Is this proposal a re-submission to the Miami CTSI KL2 Awards? (see Section 3 of the application)

⊗ No

B. SCORED REVIEW CRITERIA
1. Candidate
2. Research Plan
3. Mentoring Plan
4. Education Plan

The CTSI Pilot Awards Program utilizes the NIH 9-point rating scale (1=exceptional to 9=poor).

In assigning the scores, it is important that you consider the FULL RANGE of the rating scale . The scoring descriptors are displayed in each drop-down list.

1. CANDIDATE

CANDIDATE - STRENGTHS

Extensive experience within an HIV care context for women - knows the clinical context and multi-cultural patient population very well.
Has a history as a collaborator on research projects and publications and more recently beginning to develop her own publications as first author - upward trajectory of scholarly advancement
Well regarded in her department and among her collaborators/mentors
Data management and analysis skills and experience
With excellent set of mentors/collaborators has strong potential

CANDIDATE - WEAKNESSES ...

CANDIDATE - REVIEW SCORE - Use the FULL RANGE of the rating scale

⊗ 3- High Impact- EXCELLENT- Very strong with only some minor weaknesses

2. PROPOSED RESEARCH PLAN (Section 5 of the application)

- Significance: Is the research plan significant and does it address an important problem? Is the research plan consistent with the mission of the CTSI in terms of culturalized health sciences or to a disease issue of special concern in South Florida's unique racial/ethnic/sociocultural context? Is the research translational?

- Innovation: Is the project original and innovative?

- Approach: Are the conceptual, design, methods and analyses adequately developed, well-researched and appropriate to the aims of the proposed research project.

- Analysis Plan: Is the analysis plan on how to collect, analyze and interpret data adequate?

-Translational Plan: Does the candidate clearly describe the translational, clinical or public health impact of his/her research?

RESEARCH PLAN - STRENGTHS

Outstanding significance/potential for impact - developing a scalable m-health text-message intervention to promote adherence
Significance: Miami is a great starting place for this project but there is also potential for generalization to other geographic areas w
Innovation - personal customization of the messaging for clients and multi-lingual
2 Phase mixed methods research design, including randomization, is strong for intervention development and piloting
Appropriate approach for developing the prototype
Good access to patient population
Interdiscipinary research team, including a communications expert
Overall - very good potential for a successful project that can lead to an important advancement in the field of HIV medical adherence for minority women

RESEARCH PLAN - WEAKNESSES

No major weaknesses noted

RESEARCH PLAN - SCORE - Use the FULL RANGE of the rating scale

⊗ 2- High Impact- OUTSTANDING- Extremely strong with negligible weaknesses

3. PROPOSED MENTORING PLAN (Section 6 of the application)

Please assess:
(1) the appropriateness of the research or teaching faculty mentors' qualifications in the area of this application
(2) the quality and extent of the mentors' proposed role in providing guidance and advice to the candidate
(3) the previous experience of the mentors in fostering the development of academic researchers
(4) the involvement of the mentor in the preparation of this proposal

MENTORING PLAN - STRENGTHS

Strong plan for building on current skill set
Great mentorship team: Primary Mentor, Dr. Safren, has extensive relevant expertise and is also someone with whom she has not worked until very recently, so brings fresh mentorship. Secondary mentors - Dr. Scott and Prado - provide outstanding complementary expertise
Weekly meetings with the primary mentor as part of his research team, and monthly with co-mentors
Good support from department chair for protected time

MENTORING PLAN - WEAKNESSES

No significant weaknesses noted

MENTORING PLAN - SCORE - Use the FULL RANGE of the rating scale

⊗ 2- High Impact- OUTSTANDING- Extremely strong with negligible weaknesses

4. PROPOSED EDUCATION PLAN (Section 7 of the application)

Will this plan contribute to the scientific development of the scholar?
Please assess:
(1) the appropriateness of the content, the proposed duration of the career development plan to achieve scientific and academic independence for the scholar
(2) the usefulness of the plans for enhancing the academic skills as described in the career development plan.
(3) the likelihood that the career development plan will prepare the scholar to conduct research that will be informative/transformational concerning the biological, behavioral or sociocultural factors that influence susceptibility, progression or outcome of diseases that disproportionately affect South Florida's highly diverse racial/ethnic populations.
(4) the likelihood that the career development plan will prepare the individual for an academic career involving culturalized health sciences.

EDUCATION PLAN - STRENGTHS

Attending biostats and translational science courses
Good mix of live and online courses
Should result in advancement of research skills

EDUCATION PLAN - WEAKNESSES

Would be good to have some workshops/coursework on e-health and intervention development

EDUCATION PLAN - SCORE - Use the FULL RANGE of the rating scale

⊗ 3- High Impact- EXCELLENT- Very strong with only some minor weaknesses

C. ADDITIONAL REVIEW CRITERIA (NOT SCORED)
1. Addressing health disparities
2. Adequacy of Responsible Conduct of Research
3. Adequacy of Budget
4. Adequacy of Research Environment
5. Alignment with Medical School Strategic Plan

1. Does this proposal adequately address culturalized health or health disparities? Please provide a brief explanation to your response.

Absolutely - innovative personalized approach well based upon extensive cultural expertise of the team but also getting input from client population.

2. RESPONSIBLE CONDUCT OF RESEARCH (Section 7 of application)

Is the plan for providing formal and informal instruction in the bioethics, scientific integrity and the responsible conduct of research adequate?

⊗ Yes

3. BUDGET & BUDGET JUSTIFICATION (Section 8 of the application).

Is the budget acceptable as proposed?
NOTE: the proposed budget is a preliminary budget for $30K for research costs only. A detailed budget will be provided if the proposal is awarded.

⊗ Yes, the budget is acceptable as proposed.

4. ENVIRONMENT

Is the environment (academic, research and educational) described in the application conducive to the candidate achieving status as an independent investigator with capacity to prepare an NIH grant by the end of the funding period?
Is there adequate space and support for the proposed research project?

⊗ Yes

5. ALIGNMENT WITH MEDICAL SCHOOL STRATEGIC PLAN

Based on your review, is this project in alignment with the Medical School Research Strategic Plan? The link to the Medical School Research Strategic Plan visual overview is provided in the next field.

Please provide a short explanation to your response.

Well aligned with Miller School Strategic Plan for research - strong health disparities focus in the area of HIV/AIDS

D. SUMMARY SCORE

SUMMARY SCORE

Based on your overall review of this candidate's application, and your evaluation of the proposed research, education and mentoring plans in particular, please provide your Summary Score.
Note: the Summary Score does not have to be the average of the research, education and mentoring plans scores.

Use the FULL RANGE of the rating scale

⊗ 2- High Impact- OUTSTANDING- Extremely strong with negligible weaknesses

E. ADDITIONAL COMMENTS (Optional)

Additional comments and recommendations to applicant

,,, on this outstanding application.

CTSI KL2 Awards FY19 - Reviewer Evaluation Form

Dear Miami CTSI KL2 Reviewer,

Thank you for accepting to review this proposal.

Please submit your reviews by September 17, 2018.

If you have any question regarding this award, or the evaluation form, please contact ...

The RFA and Instructions are available on the CTSI website:
http://www.miamictsi.org/researchers/funding-opportunities/ctsi-k12-program-awards

Best regards,

The CTSI Team

Response was added on 09/10/2018 5:53pm.

CONFLICT OF INTEREST

The following are considered potential conflicts for reviewing grant proposals:
• You have a financial and/or professional relationship with the applicant
• The funding decision on any application would benefit or affect you directly
• You feel there may be a perception of conflict
• You are a current collaborator, or have collaborated/published with the applicant in the last two years.

Note: You may review applications within the same department as long as none of the above conditions apply.

By checking the box below, you confirm that you do not have any conflict of interest in reviewing this proposal

☒ I confirm

SECTION 1: REVIEWER'S INFORMATION

SECTION 2: Miami CTSI KL2 AWARD APPLICANT INFORMATION

Applicant's Name (Last,

First) ...

Department

...

Collaborator(s) on this project

Proposal Title

A Multi-Lingual, Culturally-Competent mobile Health Intervention to Reduce Medical Mistrust, Stigma, and Improve Treatment Adherence among Women Living with HIV/AIDS

SECTION 3: REVIEW

The focus of the KL2 award program of the Miami CTSI is to help outstanding early career investigators establish academic careers involving important translational, transformational research programs examining disease processes or outcomes related to health disparities.

Prior to proceed with this review, please read carefully the review criteria for the Miami CTSI KL2 Awards listed below:
• Compliance with all eligibility requirements (the administrative review is completed)
• Commitment and potential for a career in clinical and/or translational research.
• Commitment of the necessary resources and protected time from the primary department.
• Scholarship, as assessed from biosketch, letters of reference, and past academic performance.
• Evaluation of the proposed research project (scientific merit, potential clinical importance, scholar's ability to execute the research plan, available resources, likeliness of successful completion).
• Evaluation of the proposed mentored development plan, appropriateness and commitment of mentoring team and collaborators.
• Likelihood that the KL2 support will contribute to a successful K or R Award application (or equivalent).
• The impact of the research on improving the health of minority and underserved population.

The CTSI KL2 FY19 RFA and Instructions are attached here as one PDF.

[Attachment: "KL2 FY19 RFA & Instructions.pdf"]

A. Is this proposal a re-submission to the Miami CTSI KL2 Awards? (see Section 3 of the application)

⊗ No

B. SCORED REVIEW CRITERIA

1. Candidate

2. Research Plan

3. Mentoring Plan

4. Education Plan

The CTSI Pilot Awards Program utilizes the NIH 9-point rating scale (1=exceptional to 9=poor).

In assigning the scores, it is important that you consider the FULL RANGE of the rating scale . The scoring descriptors are displayed in each drop-down list.

1. CANDIDATE

CANDIDATE - STRENGTHS

Investigator. The PI has had significant training and experience in research and data/technology, has been appointed to the faculty as assistant professor in OB/GYN, and is appropriate to transition to being a junior investigator. Her background meets the criteria for the CTSI K2 funding, she has worked on a variety of research programs/grants and with the OBGYN team for many years. She has produced a well- developed application with linkages to the requirements of research (theory, model, aims, assessments and intervention inter-linked). Her letters from her mentor, chair and collaborator are very strong.

CANDIDATE - WEAKNESSES

The PI has limited training in behavioral research, but this is offset by her pending training and mentoring proposed.

CANDIDATE - REVIEW SCORE - Use the FULL RANGE of the rating scale

⊗ 2- High Impact- OUTSTANDING- Extremely strong with negligible weaknesses

2. PROPOSED RESEARCH PLAN (Section 5 of the application)

- Significance: Is the research plan significant and does it address an important problem? Is the research plan consistent with the mission of the CTSI in terms of culturalized health sciences or to a disease issue of special concern in South Florida's unique racial/ethnic/sociocultural context? Is the research translational?

- Innovation: Is the project original and innovative?

- Approach: Are the conceptual, design, methods and analyses adequately developed, well-researched and appropriate to the aims of the proposed research project.

- Analysis Plan: Is the analysis plan on how to collect, analyze and interpret data adequate?

-Translational Plan: Does the candidate clearly describe the translational, clinical or public health impact of his/her research?

RESEARCH PLAN - STRENGTHS

Research Plan
Specific Aims. Aims are clearly outlined and the background establishes the scientific justification and rationale for the study.
Significance. References pregnant and prenatal women, as the application is targeting women in this group as well as in general. Proposes that stigma can be a manifestation of medical mistrust.
Innovation. Offers good justification as to the innovation of the project (mHealth, women, languages, targeted culturally tailored messages). MHealth is a widely used strategy for reaching patients and as such is not innovative. However, use of multiple languages and the specific population of Miami (multicultural women with HIV) appears innovative, and in the current paradigm using tailored messages would be considered innovative and culturally tailored. Despite the fact that it uses messages drawn from the STEP-AD study, the concept itself is novel. Tailoring here is conceptualized as patient-centered and addressing the individual's HIV barriers within the context of multicultural women living with HIV - WLWH.
Approach
The application suggests that culturally competence is conceptualized as using tailored messages that will reduce stigma and medical mistrust (and increase resilience); these messages will be developed through a series of focus groups made up of diverse types of patients. The application outlines the entire approach very well.
Eligibility criteria addresses patients non-adherent by visit and viral load or medication uptake.
Roles of study team are clear and appropriate. It is not clear if the same person is conducted recruitment and assessments.
Phases outline Phase 1, focus groups, attendance, function. (How will the data be selected/distilled/coded/ into messages is address in ANALYSIS) Phase 2, outlines the elements of the data collection during the trial of the system. Phase 2 includes information on the intervention elements, instruments, power issues (not required).Challenges and alternative strategies are included. Overall, brevity is likely required in this application due to the 6 page limitation, and the applicant covered almost all issues.

RESEARCH PLAN - WEAKNESSES

Preliminary Work. Opening paragraph outlines disparities at the clinic on attendance, followed by detail on the clinic at UM and the participation of partners with regard to PrEP - Unclear how this relates to the current study.
Phase 2: Missing: are focus group attendees eligible for the study conditions, also? The measure for ADHERENCE is not included - how is the study assessing medication uptake? Self report? Pharmacy fill? This is also missing from the MODEL of outcome variables.
Analysis Plan. It is not clear that an ANOVA can be used for each of the study outcomes, and issues such as log transformations and outliers are neglected (investigator has support on statistics?). Overall the plan is simplistic and needs development (is this part of the education plan for the investigator?) A timeline is included and while the beginning of the study (year 1) appears compressed (too many activities) the second year appears to have more time to actually complete the study and is more appropriate.

RESEARCH PLAN - SCORE - Use the FULL RANGE of the rating scale

⊗ 2- High Impact- OUTSTANDING- Extremely strong with negligible weaknesses

---

3. PROPOSED MENTORING PLAN (Section 6 of the application)

Please assess:
(1) the appropriateness of the research or teaching faculty mentors' qualifications in the area of this application
(2) the quality and extent of the mentors' proposed role in providing guidance and advice to the candidate
(3) the previous experience of the mentors in fostering the development of academic researchers
(4) the involvement of the mentor in the preparation of this proposal

---

MENTORING PLAN - STRENGTHS

Mentoring Plan.
Research Goals. Includes research activities and aims for education and training (such as biostats training, attending conferences, working with mentors, follow on from pilot study and submission of K01 and pilot awards from other bodies, e.g., CFAR)

MENTORING PLAN - WEAKNESSES

None noted

MENTORING PLAN - SCORE - Use the FULL RANGE of the rating scale

⊗ 1- High Impact- EXCEPTIONAL- Exceptionally strong with essentially no weaknesses

4. PROPOSED EDUCATION PLAN (Section 7 of the application)

Will this plan contribute to the scientific development of the scholar?
Please assess:
(1) the appropriateness of the content, the proposed duration of the career development plan to achieve scientific and academic independence for the scholar
(2) the usefulness of the plans for enhancing the academic skills as described in the career development plan.
(3) the likelihood that the career development plan will prepare the scholar to conduct research that will be informative/transformational concerning the biological, behavioral or sociocultural factors that influence susceptibility, progression or outcome of diseases that disproportionately affect South Florida's highly diverse racial/ethnic populations.
(4) the likelihood that the career development plan will prepare the individual for an academic career involving culturalized health sciences.

EDUCATION PLAN - STRENGTHS

Education Plan.
Coursework for the award is well described, and are really the essential element for this investigator to achieve independence. A plan for coursework is outlined, and as the plan develops, added activities are included.

EDUCATION PLAN - WEAKNESSES

None noted.

EDUCATION PLAN - SCORE - Use the FULL RANGE of the rating scale

⊗ 1- High Impact- EXCEPTIONAL- Exceptionally strong with essentially no weaknesses

C. ADDITIONAL REVIEW CRITERIA (NOT SCORED)
1. Addressing health disparities
2. Adequacy of Responsible Conduct of Research
3. Adequacy of Budget
4. Adequacy of Research Environment
5. Alignment with Medical School Strategic Plan

1. Does this proposal adequately address culturalized health or health disparities? Please provide a brief explanation to your response.

This innovative project combines mHealth, women, languages, targeted culturally tailored messages using multiple languages and the specific population of Miami (multicultural women with HIV), and in the current paradigm will address health disparities among women living with HIV. The concept is patient-centered and can reduce health disparities by addressing HIV barriers within the context of multicultural women living with HIV.

2. RESPONSIBLE CONDUCT OF RESEARCH (Section 7 of application)

Is the plan for providing formal and informal instruction in the bioethics, scientific integrity and the responsible conduct of research adequate?

⊗ Yes

3. BUDGET & BUDGET JUSTIFICATION (Section 8 of the application).

Is the budget acceptable as proposed?
NOTE: the proposed budget is a preliminary budget for $30K for research costs only. A detailed budget will be provided if the proposal is awarded.

⊗ Yes, the budget is acceptable as proposed.

4. ENVIRONMENT

Is the environment (academic, research and educational) described in the application conducive to the candidate achieving status as an independent investigator with capacity to prepare an NIH grant by the end of the funding period?
Is there adequate space and support for the proposed research project?

⊗ Yes

5. ALIGNMENT WITH MEDICAL SCHOOL STRATEGIC PLAN

Based on your review, is this project in alignment with the Medical School Research Strategic Plan? The link to the Medical School Research Strategic Plan visual overview is provided in the next field.

Please provide a short explanation to your response.

The grant application focuses on HIV/AIDS, an element/pillar of the UM strategic plan.

Miller School of Medicine Strategic Plan - PowerPoint slide

[Attachment: "MSOM Strategic Plan slide.pptx"]

D. SUMMARY SCORE

SUMMARY SCORE

Based on your overall review of this candidate's application, and your evaluation of the proposed research, education and mentoring plans in particular, please provide your Summary Score.
Note: the Summary Score does not have to be the average of the research, education and mentoring plans scores.

Use the FULL RANGE of the rating scale

⊗ 2- High Impact- OUTSTANDING- Extremely strong with negligible weaknesses

E. ADDITIONAL COMMENTS (Optional)

Additional comments and recommendations to applicant

Very nicely developed and strong!

Have you joined the UM Internal Awards Reviewer Pool?

⊗ Yes
